# Book Reviews

**Published:** 1841-09

**Authors:** 


### Bibliographical Notices.

*Researches on the Development, Structure and Diseases of the Teeth.* By ALEXANDER NASMYTH, F. L. S., F. G. S., Member of the Royal College of Surgeons, Fellow of the Royal Medical and Chirurgical Society. LONDON, 1839, pp. 165, 8vo.

THE lengthy and very able article copied into our January and February numbers, from the "*Boston Medical and Surgical Journal,*" "on the Development and Structure of the Teeth," supersedes the necessity of a review from us, of the above work. We cannot, however, let the opportunity pass, without expressing the high gratification which we derived from reading it. From beginning to end it is replete with interesting and valuable information, comprising the results of the researches on the development and structure of the teeth of nearly every writer of any eminence on this branch of odontology.

The volume before us is only an historical introduction to the main work. The remainder has not yet been published, but judging from the learning and research exhibited in this, we anticipate a treatise of very great excellence, and wait with no little impatience its appearance.

Mr. Nasmyth is a man of no ordinary ability, and from the attention with which he has evidently investigated the subject of his treatise, is eminently qualified to do justice to it.

"The teeth," says Mr. Nasmyth, "may be regarded in the first place as the armoury of the mouth; and in the second, as the instruments by which the process of assimilation is commenced. They assist in seizing, dividing, tearing, and masticating the substances which the diversified surface of the earth, the fathomless depths of the ocean, and the boundless expanse of atmosphere, afford in infinite variety as materials for building up the physical frame-work of animated existence." Again, he remarks, "in the sequel to the present work it will be shown that, besides the general conformation and external appearance of the teeth, another distinctive peculiarity by which the Almighty has characterized the different members of the animal kingdom, exists in their beautiful internal, minute, and hitherto hidden organization, which further demonstrates in a most striking manner the harmony of all created existence."

Mr. Nasmyth promises that the next part of his work shall contain an account of the development and structure of the teeth. The present volume does not inform us what his views are upon these subjects, except that he does not believe in the tubular doctrine as maintained by Retzius and others. The opinion, therefore, that has been advanced, that, when a tooth has been filled, the "mouths of these vessels" deposite a bony layer around the filling, is objected to by him as being opposed to any known facts; and besides, he regards the "existence of a system of opened mouthed vessels ready to pour out fluid of any kind to a greater or less extent," as incompatible with the character of a tooth and the offices it has to execute. The admission of this, would at once be, the endowing of dental bone with recuperative powers, which it is well known it does not possess, and if we deny it, we must reject the doctrine that the decay of the organs is the result of an increased action of their vessels. For, if they be not possessed with restorative powers, it seems no more than fair to presume that they are not endowed with powers whereby they can effect their own destruction. The operations of the economy of other parts of the animal organization, would, at any rate, seem to justify this conclusion, and which, when taken in connection with the fact, as has been shown, time after time, that inflammation of the bony structure of the teeth is not necessary to caries in them, the correctness of the assumption hardly admits of doubt.

But to return—"In man," remarks Mr. N. "the mouth is as important as in the lower animals, for the performance of functions which are common to both, and has also other offices corresponding to the intellectual qualities, with which he is exclusively endowed. With him, too, it is the porch of assimilation, but with him alone it serves for the expression of mental operations—the elevated characteristic of his race. He not only performs mastication by its means, but accomplishes with its aid the emission of articulate sounds; communicates his ideas to his fellow-men, receiving information conveyed in the same manner in return; and thus are means furnished for the infinite progress and improvement of mankind. By the endowment of speech man is enabled to command and direct the beings around him, and to bring to bear on a single point the will of a whole race. The teeth are of essential importance in the exercise of this faculty, for the eloquence of the statesman would be powerless, and the force of his arguments unavailing, if they were deprived of the sonorous utterance imparted to them by these organs, without which, too, the warrior's exhortation and command must lose their electrifying influence."

Again, he says, "I have endeavoured to conduct my investigations on a scale which I conceived commensurate with the importance of the subject; and in detailing the results which I have arrived at, I shall give as careful a description as possible of the nature of the experiments, dissections, or observations which lead me to them. The substance of what I have to offer will go to prove, I think, a greater degree of simplicity and uniformity, than has hitherto been suspected in the organization of these structures."

Although the formation, structure and diseases of the teeth have long engaged the attention of men of learning and high physiological attainments, there yet remains much to be learned; and it is gratifying to perceive that researches into this department of science are still being made, both in Europe and America, and that, too, by men, and with a zeal that promises the most useful and valuable results.

The microscopic examinations of the teeth by Retzius, though they have not resulted in a satisfactory explanation of the manner in which the calcareous molecules in dental bone are arranged, have certainly added much to the stock of information that before existed on the subject, and Mr. Nasmyth has done well in presenting the English reader with a translation of his papers, containing an account of them.

Besides giving a view of the researches of modern writers on odontology, the progress of the science from the earliest periods of which any authentic account of it has reached us, is rapidly and at the same time very accurately traced by the accomplished author of the work under notice. Commencing with the allusions made by *Herodotus* to the manner in which medicine was practised in ancient Egypt, when its practitioners were divided into classes, each restricted to the treatment of diseases of the particular part of the body assigned it, some taking charge of those of the eyes, others those of the teeth, &c. &c. he says,

"Of the views entertained of the physiology and pathology of these organs in Greece, before the time of *Hippocrates,* we know but little, except that they are embodied in the works of the latter. *Homer* calls the teeth, 'little barriers opposed by nature to the unruliness of the tongue, and to the abuse of speech.' "

The crude and imperfect notions of *Hippocrates* in regard to the origin and formation of the teeth is next noticed. The opinions of *Aristotle, Aretœus, Galen, Ætius,* and *Vesalius,* are then briefly alluded to, the last of whom, though styled, and very justly so, the restorer of anatomy, does not seem to have carried his anatomical researches to the dental organism. The views of *Eustachius,* the first who seems to have studied the teeth with much care or attention, are remarked upon somewhat more at length. Passing this author, *Ambrose Paré,* is the next that claims his attention, and from whose celebrated work, among other things he quotes the following. "The teeth differ from the other bones, because they have action while they chew the meat, because being lost they may be generated, and for that they grow as long as the party lives; for otherwise, by the continual use of chawing, they would be worn and wasted away by one another." This belief is entertained by a writer of little less celebrity and of a much later date. We allude to that distinguished physiologist *Richerand.* He asserts that the teeth waste by friction and grow and repair themselves. Among the older writers, many very singular and absurd notions were entertained in regard to the teeth. Gibson, an anatomist of the seventeenth century, observing that the temporary teeth had no roots when they were shed, supposed they remained in the sockets, and to quote his own words, "which being like seeds for the new ones, by degrees grow up above the gums, to supply the place of that which was fallen off." Numerous other equally erroneous and fantastic ideas pertaining to the manner of the formation of the teeth, and to their diseases and treatment, prevailed at and even subsequent to this time.

Cotemporary with the author last mentioned, and others equally uninformed on the subject of the teeth was *Leeuwenhoek,* who, as is seen by his publications in the *Philosophical Transactions,* for 1687, seems to have studied it with much attention, and, it is a matter of surprise that, the promulgation of the result of his numerous and highly interesting researches did not attract at the time more notice than they seem to have done.

Returning however, to the writings of that celebrated French surgeon, *Ambrose Paré;* we will quote from the work before us, one passage on the insertion of artificial teeth, and though the method described by him of doing it was long ago abandoned by all well informed practiciens, it is within the recollection of many, not yet gray in the profession, when it was followed by not a few engaged in the calling of the dentist. "Teeth," says he, "artificially made of bone or ivory may be put in the place of those that are wanting; and they must be joined one fast unto another, and also so fastened unto the natural teeth adjoining that are whole; and this must chiefly be done with a thread of gold or silver, or, for want of either, with a common thread of silk or flax, as it is declared at large by Hippocrates, sect. ii. *lib. de art. sent.* xv."

A brief expose of some of the views of *Scaliger, Kerkring, Malpighi, Leeuwenhoek, Duverney* and *Winslow,* next follows. Continuing the historical account of the progress of the science of odontology, the author, after noticing the writings of *Bertin, Herissant, Bourdet,* and *Jourdain,* on the subject, arrives at the work of the famous English anatomist and surgeon, *John Hunter,* on the teeth. This treatise is noticed more at length, and in doing which, Mr. Nasmyth takes occasion to point out the great similarity that exists between the opinions of its author and his distinguished predecessor, Ambrose Pare. A comparison of the following passages are instituted.

"Pare, in the 30th chapter of his 10th book, recommends a hot iron, or cotton dipped in oil of vitriol to be applied in case of tooth-ache, 'in order to burn the nerve which is inserted into their roots.' This does not much differ from Mr. Hunter's treatment, as we learn from the following passage: 'The nerve may be burned, that this may have the desired effect, it must be done to the very point of the fang.' Again, 'Either of the concentrated acids, such as those of vitriol, nitre, or sea-salt, introduced as far as possible, is capable of destroying its soft parts, which most probably are the seat of pain.'"

A comparison of a number of other passages from the works of these two eminent men is made by Mr. N. which, it is not necessary for us to copy.

Continuing the subject, condensed notices and brief extracts from the writings of *Bichat, Blandin,* Blake, Fox, Bell, Koecker, Jobson, and Waite, interspersed with quotations from some of the works previously presented to the reader, succeed to the historical sketch of Hunter's treatise on the natural history and diseases of the teeth.

Having rapidly traced the progress of the science thus far, the works of *Baron Cuvier, Serres, F. Cuvier, Rousseau, Weber, Tenon, Purkinje,* and *Müller,* are the next that engage the consideration of the author, and are passed over less hastily than the preceding ones. Ariving at this point in his narrative, the researches of Professor *Retzius,* of Stockholm, next receive his attention, and to these a large portion of his work is devoted.

We should be glad, had we time, to give a more extended notice of this highly interesting work; of its value however, the reader will be able to form some idea from what we have already said, and as it is presumed that every member of the profession will soon endeavour to possess it, a more elaborate description, is perhaps unnecessary.—*Balt. Ed.*

*A Practical Treatise on the Human Teeth, showing the causes of their Destruction, and the means of their Preservation.* By William Robertson, with plates. London, Hayward & Moore, 1839, pp. 205, 8vo.

The unphilosophical and erroneous doctrine, that caries of the teeth is the result of inflammation of their bony structure, is, we are gratified to learn by the above and other European publications on the teeth, not universally held by our professional brethren on the other side of the water. Its advocates, we believe, are becoming less numerous every day, and opposed as it is to every fact connected with the disease, it is somewhat remarkable that any one in the daily habit of observing it in its different stages could have supposed it to proceed from this cause. We call the doctrine unphilosophical and erroneous, because it has been shown, and that too, by more than one, and by facts that cannot be disproved, that caries of the teeth is produced by other causes than this, and if they are capable of giving rise to the disease, we do not conceive it the part of sound philosophy to ascribe its occurrence to another, the competency of which to orginate it, cannot be shown by any other evidence than mere hypothetical reasoning.

The discovery of the vascularity of the teeth, while it, on the one hand, may be said to have assisted to a more correct explanation of the phenomena of some of the diseases of these organs than had previously existed, it on the other, certainly gave rise to much error of opinion in regard to those of others. For, no sooner was the doctrine promulgated that dental bone was identical with that in other parts, than an analogy was at once supposed to exist between the maladies of the teeth and those of other osseous structures. But to have been consistent, those who maintained these views, should in the treatment of the diseases of the one, have applied the same remedies, that were recommended for those of the other, for, if their diseases be alike, their remedial indications would also be the same. But that disease of the teeth which is designated by the term caries, and this is the one upon which Mr. Robertson principally treats, bears not the slightest analogy to the disease that is called by that name in other bone; it does not possess any of its characteristics, is not dependent upon the same cause, nor is it susceptible of cure by the same remedies. It is not however, our purpose to enter upon a discussion of this subject on the present occasion, we shall therefore, at once proceed to notice the work now under consideration; and in the first place, having in what we have already said, intimated the prominent doctrine set forth in it, would remark, that, while with its author in this, we most heartily concur, we regret that he has passed unnoticed those by whom it had been before promulgated. Is it possible that he was not aware that the opinions which he has advanced in regard to the causes that produce dental caries, had been previously given, and that too, by more writers than one! if so, we can only say that he should have been more thoroughly read on the subject on which he has treated, before having himself become an author on it. We would however, notwithstanding, do him the justice to say, and we do this the more readily, because we are for the most part pleased with his work, that we do not think he was aware of the fact to which we have here alluded. From the spirit of candour, with which he seems to treat the subject, we believe that, had he been conversant with the works of those by whom the doctrine maintained by himself, had been previously advocated, he would not have assumed the credit of having originated it.

But having said thus much, we will mention from among English and American writers on odontology, the names of a few, by whom the doctrine set forth by Mr. Robertson, had been previously promulgated and we will commence with that of Dr. Samuel L. Mitchell, of New York, whose views upon this, among other subjects, are contained in a letter addressed to Thomas Charles Hope, M. D. &c. adjunct professor of chemistry in the University of Edinburg; dated October 10th, 1796, and subsequently in 1805, published in the New York Medical Repository.

In the foregoing paper is shown by actual experiments, that an acid is formed in the mouth, and supposed to be the result of a chemical decomposition of lurking particles of food and other extraneous matter about the teeth, and that it is by the union of this with their calcareous phosphate that their decay is effected.

In a memoir published in the Medical Repository, for 1813, Professor H. H. Hayden, dental surgeon of Baltimore, thus remarks:

"I shall in the next place endeavour not only to prove my position as it respects the glands, but to point out and identify what, at least in my opinion, constitutes the universal cause of the decay of the human teeth." * * * * * * * *

"Therefore I feel no hesitation in saying that the decay of the human teeth is occasioned almost universally by an acrid, or otherwise morbid secretion from the mouth and gums."

But the doctrine that the decay of the human teeth is occasioned by the action of external chemical agents was promulgated more directly to the profession by Doctor Eleazar Parmly, and by his brother Doctor L. S. Parmly, about twenty years ago, whose views as published in a treatise on the teeth, by each, were at the time noticed in several European Medical Journals. In fact, the first edition of the work of the former was published in London where the author at that time resided. From this we quote the following:

"From my own observations, I am induced to believe that caries is universally caused by the action of external agents, and therefore cleanliness, after the proper offices of the dentist are performed, is the only guard against it, but some teeth from their being of less dense structure," a circumstance which Mr. Robertson seems not to have taken into consideration, "are less capable of resisting the action of decomposing matter."*

Doctor L. S. Parmly says, "by a chemical action of those relics of food, which accidentally lodge between them, (the teeth,) a deleterious change takes place, constituting an active poison, which corrodes their structure."†

* An Essay on the Disorders and Treatment of the Teeth.

† Lectures on the Natural History and Management of the Teeth.

More recently, in 1829, the same doctrine was mantained by J. Patterson Clark, A. M. dentist, of London, in a treatise entitled, "a new system of treating the human teeth," as may be seen by the following quotation.

"When indentation, or such like inequality occurs on the surface of the teeth, the juices of the mouth there become stagnant; their properties change, and they exert a pernicious influence, aided by the putrifying particles of animal and vegetable substances which likewise necessarily lodge there."

Mr. Clark adduces many facts to prove that decay of the teeth is caused by the presence of vitiated secretions of the mouth and decomposed particles of food. We could refer to other authors who hold the same views in regard to this subject, but the opinions we have already quoted suffices our purpose.

Mr. Robertson however, has treated the subject at greater length than any preceding writer, and we must do him the credit, to say that he has maintained the position which he has assumed in regard to it, with great ability—having shown by arguments and facts, that cannot, in our opinion, be successfully controverted, that the decay of the teeth is occasioned in no other way than by the action of external corrosive agents. The clear and lucid manner in which he has set forth this doctrine, and the conclusive proofs he adduces in its support, cannot fail to carry conviction of its truth to the minds of all who read his book, and though intended rather for the general reader, than the dental practitioner, the latter should by all means possess it. It contains much valuable information. On the pathology of dental caries, we regard it as a very good work and can therefore, without hesitation commend it to the profession. The author has unquestionably paid much attention to that disease, and what he says of it, seems more the result of personal observation and experience, than opinions formed from other writers. In fact, the only works with which he appears to be at all familiar, are those of Hunter, Fox, and Bell, and the doctrine set forth in these in regard to the decay of the teeth he proves to be erroneous.

From his introductory remarks we make the following quotations. "It may be safely asserted, that none of the organs of the human body are so often the subjects of disease as the teeth; none, the disease of which are so little understood, and yet, when rightly comprehended, none, more completely under individual control."

This opinion is in part correct, but not wholly so. The teeth beyond question are more subject to disease than any of the other parts of the body, and can with greater certainty be controlled; but, that they are less understood is so far from being the fact that they are probably much better.

Alluding to Messrs. Fox and Bell, he says, "each of these has promulgated a theory on the nature and origin of decay of the teeth, and both with the professed object of supplying the deficiency of Mr. Hunter." In noticing the theories of these scientific authors, he a little further on thus remarks, "The mistaken notions which have been advanced upon this subject, it is of the more importance to correct, because they erroneously suppose the proximate cause of decay to be inflammation of the internal substance of the tooth, commencing according to the theory of Mr. Bell, upon the surface of the bone, within the enamel; and, according to the doctrine of Mr. Fox, originating in the internal membrane; and consequently, the primary seat of the disease being in the interior of the tooth, it does not discover itself externally until it has made considerable ravages in the internal substance of the organ; an error of the greater importance because it leads the public in general, to whom the influence of the opinions of eminent men naturally extends, to neglect the teeth during the first stages of decay when its progress might be arrested, and to withhold their attention until acute suffering and irremediable mischief have been produced."

In the second chapter of his work the theories of these two gentlemen are examined more at length. The erroneous views entertained by Mr. Fox of the cause of dental caries is attributed by Mr. Robertson to his having "compared the teeth with other bones," and to the opinion held by him that a "similarity of structure and organization existed in both;" and in support of this assertion quotes from his treatise on the teeth, the following passage.

"That this is the proximate cause of caries" says Mr. Fox, "appears to be highly probable, by remarking that a caries of other bones is caused by a separation of those membranes which cover them. Thus a separation of the periosteum will cause a death of the tibia or that of the pericranium a caries of some part of the bones of the head."* "This opinion," says our author, "he considers, is confirmed by comparing the symptoms which accompany inflammation in a bone with those which are occasionally felt by persons in their teeth, previously to any appearance of caries."

Arguing from these premises, Mr. Robertson contends, and very justly so too, that tooth-ache, a never failing consequence of inflammation of the lining membrane of a tooth, would always precede caries, which common experience and daily observation proves to be not the fact; on the contrary, that inflammation of this membrane and pain, is rather the result of previous decay, and hence the danger of neglecting the teeth until admonished by pain, that disease is committing its ravages upon them; when, as it not unfrequently happens, the devastations committed by it are such, as to render the skill of the dental surgeon inadequate to the reparation of the injury.

Mr. Robertson furthermore contends that were the theories of Mr. Fox and Mr. Bell true, all the teeth would be equally subject to caries, which according to these gentlemen's own showing is not the fact.

Agreeably to Mr. Bell's theory, the surface of bone most remote from the internal membrane, he maintains, would be the part most liable to be first attacked by the disease, when, in reality the very reverse is true.

In the third chapter the author adduces proofs in support of the doctrine that the decay of the teeth is occasioned by the action of chemical agents, these, our limits will not permit us to notice. We will, however, quote the first paragraph, the views of the author being therein set forth.

"Upon examination," says he, "it will be found that there are fissures formed in the enamel of the teeth, in consequence of the irregular distribution of that substance upon their surfaces; and that there are also interstices produced by the crowded position of the teeth, and the irregularity of their

* Inflammation and separation of the lining membrane of a tooth may cause the death of the organ, and still not give rise to caries in it—a distinction which Mr. Fox, in this place, seems to have overlooked.

shape: in these situations particles of food are retained, which undergo a process of decomposition and acquire the property of corroding, disuniting, and thereby destroying the earthy and animal substance of which the teeth are composed. That this is the cause of the destruction of the teeth, commonly called 'caries,' and that it is not the result of inflammation, either in the membrane or the bone of the tooth, will appear obvious from facts which will be produced, and which I have daily witnessed during a long course of practice."

The only circumstance that he seems to have noticed as constituting a difference in the liability of different teeth to decay, is that which is found in their shape; but there is another, and that is the density of the organs. In the ratio as they are hard or soft is their liability to be acted upon by external corrosive agents increased or diminished; and it is worthy of remark, that dense teeth have fewer lodgments for extraneous matter than those that are soft. Teeth that are hard, and well arranged are less liable to be attacked with caries, than those that are soft and form an irregular arch. Our views however, having been given somewhat at length in another place,* it is unnecessary to repeat them here.

The more effectually to establish the theory of the decay of the teeth which he advocates, he denies the vascularity of the organs. This we conceive was unnecessary, and had he investigated their structure with as much attention as he seems to have done that of their diseases, he would hardly have fallen into this error, we call it an error because the contrary has been satisfactorily proven, anatomical research has long since placed this matter at rest. We have in our possession at this time, two upper incisor teeth, which were presented to us by Mr. F. H. Clark, surgeon dentist of this city, taken from a person who had died of asphyxia,† that are tinged with red. We have also two thin slices of bone taken from the root of an inferior molaris, extracted from the mouth of a boy about thirteen years of age, in each of which, by the aid of a microscope magnifying 675, a vessel injected with red blood can be distinctly seen. These, we exhibited to the class of the Baltimore College of Dental Surgery, but a few months since, and they have also been examined by several medical gentlemen.

But notwithstanding the erroneous views which Mr. Robertson entertains on some of the subjects upon which he treats, his opinions are, as we have before said, for the most part correct. He has one very excellent chapter on the bad effects that result from the retention of teeth, so much diseased that they cannot be restored, in the mouth. We wish it could be read by every one. Succeeding to this, he has one on the loss of the teeth from tartar and from inflammation of the dental periostum.

The work being designed principally for the general reader, the author enters but little into practical detail.—*Balt. Ed.*

* See the Dental Art, a practical treatise on Dental Surgery.

† See Professor Hayden's article on this subject in the 9th and 10th numbers of this Journal, vol. 1, p. 212.

*Popular Treatise on the Structure, Diseases, and Treatment of the Human Teeth, with an illustration of the present state of Dental Mechanism.* By J. L. Murphy; London; Whittaker & Co. 1837, pp. 203, 12mo.

Information concerning the means necessary for the preservation of health cannot be too widely disseminated; but in the reception of opinions on a subject involving so much of human comfort and happiness, we should be careful to be influenced by such only as come from accredited sources. We should endeavour, therefore, to discriminate between those that are based upon hypothesis, and those that are founded on well established facts or are the result of long, careful and accurate observation and experience. The *ipse dixit* of an individual concerning that with which, we have no evidence that he himself is thoroughly acquainted, should not be too hastily received, lest by a too easy credulity, we be lead into error, and suffer, perhaps, in consequence, irreparable injury. It is not every one who assumes to teach, that is competent to the task, or is himself well instructed.

The volume before us, is handsomely got up; the language is plain, and sufficiently abundant to a ready and full comprehension of the views of the author. The whole work is comprised in seven chapters. The relationship subsisting between the dental apparatus and other parts of the body are treated on in the first, and from which, is deduced the importance of the art of the surgeon dentist. In this chapter also, are advanced some very cogent reasons, for a general "cultivation of the knowledge of the teeth." With these, all who have the care of children should be acquainted, and were they to properly feel their force, much of the suffering and disease that results from inattention to these organs, would, we are persuaded, be avoided.

The principles of practice which Mr. Murphy lays down and recommends are not in every particular, such as are recognized by scientific and experienced practitioners of dental surgery. The empirical practice of filling teeth with "*metallic cements*,"—the principle ingredient of which is crude mercury, we regret to say, he recommends in many cases. The deleterious effects of these articles upon the teeth and mouth generally, are too well known in this country to need comment. It may be they have not had as much experience in England with them as here. We hope they have not, and that they never will have, for, of all the articles, of which we have any knowledge, that has ever been employed for stopping teeth, this is decidedly the most objectionable; and we want no better evidence of a man's ignorance of this nice and difficult operation, than for him to use, or recommend the use of this poisonous compound. Tin or even lead is incomparably better.

We are inclined to believe, that Mr. Murphy has had little practical experience in the treatment of the diseases of the teeth, from the fact, that he supposes any one can go through with the manipulations pertaining to that, of at least, many of their maladies. With a view to convince his readers of this, he says, "Every art is calculated in some degree to be practised by all, and each has its simpler operations, the knowledge of which, serving as a correct guide to their performance, cannot but be beneficial." With most of the arts, this may be true, but not with that of the treatment of the diseases of the teeth. The means necessary to their prevention, can, and should be thoroughly understood, and regularly employed by all. None, except such as have been thoroughly instructed and long exercised in the treatment of the diseases of these organs are capable of arresting them.—*Balt. Ed.*

### Recent Publications.

